# Candidate genes for monitoring hydrogen peroxide resistance in the salmon louse, *Lepeophtheirus salmonis*

**DOI:** 10.1186/s13071-020-04211-1

**Published:** 2020-07-10

**Authors:** Celia Agusti-Ridaura, Marit Jørgensen Bakke, Kari Olli Helgesen, Arvind Y. M. Sundaram, Sigrid Jørgensen Bakke, Kiranpreet Kaur, Tor Einar Horsberg

**Affiliations:** 1 Faculty of Veterinary Medicine, Sea Lice Research Centre, Norwegian University of Life Sciences, 1433 Aas, Norway; 2 Department of Epidemiology, Norwegian Veterinary Institute, Pb. 750 Sentrum, 0106 Oslo, Norway; 3 Department of Medical Genetics, Oslo University Hospital, 0450 Oslo, Norway; 4 Department of Geosciences, The Faculty of Mathematics and Natural Sciences, University of Oslo, 0371 Oslo, Norway; 5 Aker Biomarine Antarctic AS, P.O. Box 496, 1327 Lysaker, Norway

**Keywords:** H_2_O_2_ resistance markers, Sea lice, RNAseq, Catalase, Aquaporin

## Abstract

**Background:**

Hydrogen peroxide (H_2_O_2_) is one of the delousing agents used to control sea lice infestations in salmonid aquaculture. However, some *Lepeophtheirus salmonis* populations have developed resistance towards H_2_O_2_. An increased gene expression and activity of catalase, an enzyme that breaks down H_2_O_2_, have been detected in resistant lice, being therefore introduced as a resistance marker in the salmon industry. In the present study the aim was to validate the use of *catalase* expression as a marker and to identify new candidate genes as additional markers to catalase, related to H_2_O_2_ resistance in *L. salmonis*.

**Methods:**

A sensitive and an H_2_O_2_ resistant laboratory strain (P0 generation, not exposed to H_2_O_2_ for several years) were batch crossed to generate a cohort with a wide range of H_2_O_2_ sensitivities (F2 generation). F2 adult females were then exposed to H_2_O_2_ to separate sensitive and resistant individuals. Those F2 lice, the P0 lice and field-collected resistant lice (exposed to H_2_O_2_ in the field) were used in an RNA sequencing study.

**Results:**

*Catalase* was upregulated in resistant lice exposed to H_2_O_2_ compared to sensitive lice. This was, however, not the case for unexposed resistant P0 lice. Several other genes were found differentially expressed between sensitive and resistant lice, but most of them seemed to be related to H_2_O_2_ exposure. However, five genes were consistently up- or downregulated in the resistant lice independent of exposure history. The upregulated genes were: one gene in the DNA polymerase family, one gene encoding a Nesprin-like protein and an unannotated gene encoding a small protein. The downregulated genes encoded endoplasmic reticulum resident protein 29 and an aquaporin (*Glp1_v2*).

**Conclusions:**

*Catalase* expression seems to be induced by H_2_O_2_ exposure, since it was not upregulated in unexposed resistant lice. This may pose a challenge for its use as a resistance marker. The five new genes associated with resistance are put forward as complementary candidate genes. The most promising was *Glp1_v2*, an aquaglyceroporin that may serve as a passing channel for H_2_O_2_. Lower channel number can reduce the influx or distribution of H_2_O_2_ in the salmon louse, being directly involved in the resistance mechanism.
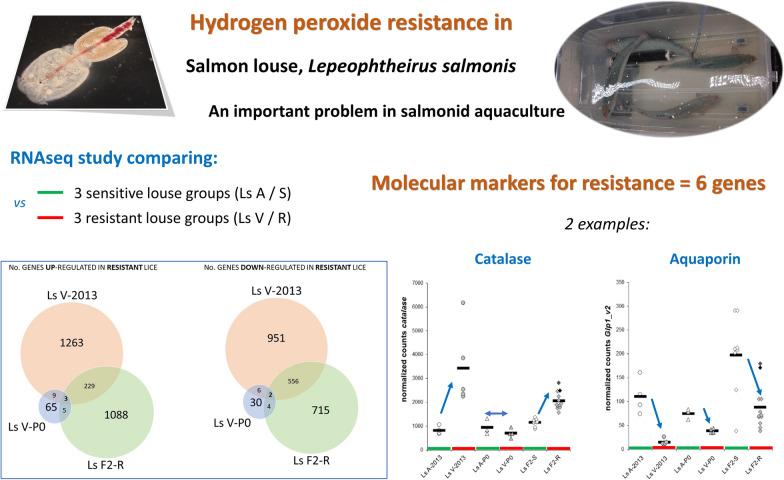

## Background

The salmon louse *Lepeophtheirus salmonis* (Copepoda: Caligidae) is one of the most important parasitic problems in the Northern hemisphere salmonid aquaculture [1, 2]. It also represents a hazard for wild salmonids [3]. Control of the parasite has historically been based on anti-lice chemicals. However, overuse, in order to keep the louse numbers below the maximum permitted levels in Norway, has led to the development of reduced sensitivity towards most of the available chemical treatments [1, 2]. Alternative mechanical and preventive methods have been developed to avoid this problem [3]. Currently, salmon lice control in Norway is based on a combination of preventive, mechanical and chemical delousing methods, as well as extensive monitoring of lice infestation and lice sensitivity to anti-lice treatments [2, 4–6].

Hydrogen peroxide (H_2_O_2_) is one of the anti-lice compounds used for controlling salmon lice infestations [7]. It was used between 1993 and 1997 in Norway as a delousing agent, but new chemicals showing better efficacy and better safety margins for fish and farm personnel replaced it. In 2009, H_2_O_2_ was reintroduced in the Norwegian salmonid farming industry [8], when reduced efficacy of other chemical treatments was identified [1]. H_2_O_2_ is also used in Norwegian aquaculture for treating the amoebic gill disease caused by *Paramoeba perurans* [9]. As a result, there was a large increase in the use of this compound in the period 2014–2016 [10]. In 2015, reports on reduced sensitivity towards H_2_O_2_ in salmon lice were published [11], and the use of this compound was limited due to reduced efficacy. Bioassays on parasites collected in the field, as well as on their descendants, showed a considerable increase in the EC_50_ values (the compound concentration affecting 50% of the parasites), confirming higher tolerance to H_2_O_2_ in these cohorts compared to parasites from a susceptible laboratory reared strain [11]. H_2_O_2_ resistance in salmon lice is an important issue not only in Norway, but also in other salmon producing countries such as Scotland [12].

In biological systems, H_2_O_2_ is a naturally occurring reactive oxygen species molecule with cytotoxic effects. It has an important function as a signalling molecule that affects a variety of processes, e.g. immune responses [13]. Several enzymes are involved in the production and regulation of endogenous H_2_O_2_. Therefore, it was not surprising to discover that catalase was involved in the mechanism providing protection to the salmon lice against H_2_O_2_ exposure, as this enzyme catalyses the breakdown of H_2_O_2_ to H_2_O and O_2_. It was shown that resistant salmon lice had higher *catalase* gene expression and catalase enzymatic activity compared to sensitive lice [14]. The expression level of *catalase* was therefore introduced as a H_2_O_2_ resistance marker in the salmon industry [15]. An accurate time-space monitoring of the sensitivity level of salmon lice to H_2_O_2_ is necessary in order to apply correct control measures. In addition, to know beforehand if the parasites are resistant is highly beneficial in order to avoid the economical, fish welfare and environmental costs of an unsuccessful treatment. Molecular methods have been demonstrated as powerful tools for monitoring the sensitivity of sea lice to chemicals [16, 17], hence the importance of improving and developing such tools for all anti-lice compounds.

In addition to the catalase enzyme, it would be expected that the lice possess additional mechanisms to protect themselves against high levels of H_2_O_2_ [12]. RNA sequencing (RNAseq) is a powerful tool to compare gene expression (as number of transcripts) between selected groups, for all genes simultaneously. This allows for the identification of genes potentially associated with such mechanisms as resistance.

The aims of the present study were to (i) validate the use of the commercially available H_2_O_2_ resistance marker (*catalase* expression), (ii) identify new candidate genes for developing molecular markers based on differential expression, and (iii) use the annotation of the candidate genes to put forward new hypotheses on the resistance mechanism for H_2_O_2_ in salmon lice.

## Methods

### Salmon louse strains

Two well-characterized laboratory *L. salmonis* strains were used in this study: Ls A, sensitive to all anti-salmon lice compounds used in Norway (tested by bioassays); and Ls V, resistant to azamethiphos, deltamethrin, emamectin benzoate and hydrogen peroxide (field reports and bioassays). Ls A was a strain originally collected on a fish farm in the northern part of Norway in 2011. Ls V was collected in October 2013, from a fish farm in mid-Norway with high anti-louse treatment pressure and reported diminished H_2_O_2_ treatment efficacy. A total of 14 anti-louse chemical treatments were performed from August 2012 to September 2013 in that farm: 6 H_2_O_2_ treatments (up until one month before the lice collection); 6 combined treatments with deltamethrin and azamethiphos; 1 treatment with diflubenzuron; and 1 with emamectin benzoate. The Ls V-2013 samples referred to in the current study were the original field samples of this strain. Ls A and Ls V strains were reared in continuous cultures at the research facilities of Solbergstrand (The Norwegian Institute for Water Research, NIVA, Drøbak, Norway), as described by Hamre et al. [18]. Both strains were maintained without any selection by medicinal compounds.

### Crossing experiment and bioassays

In order to obtain lice samples from the same generation and with a range of H_2_O_2_ sensitivities, a batch crossing experiment was designed. The experiment was performed as described by Bakke et al. [19] in 2015. Briefly, 2 Atlantic salmon (1 fish per tank) were infested with approximately 50 Ls A copepodids each and another 2 fish (1 fish per tank) with the same number of Ls V copepodids to produce the parental generation (P0). All salmon lice were collected from all fish when the lice were in the pre-adult II stage, before mating occurred. Then 10 pre-adult II Ls A females and 10 pre-adult II Ls V males from the P0 generation were distributed equally on 2 fish kept in individual tanks, to produce the F1 generation of family group 1. The same procedure was used to produce the F1 generation of family group 2, only with opposite sex from each strain, i.e. females from Ls V and males from Ls A. All P0 lice from both family groups were preserved in RNAlater (Sigma-Aldrich, MO, USA) after removal of the egg strings which were set aside to hatch. After ~ 24 h at room temperature, the preserved samples were stored at − 80 °C. Four fish were infested with copepodids from the F1 generation: 2 fish with copepodids from the family group 1 and 2 fish with copepodids from the family group 2. The lice developed to the adult stage, mated, and egg strings for the F2 generation were collected. Approximately 500 copepodids from each of the family groups 1 and 2 (F2) were used for infestation of 8 Atlantic salmon for each family group, with the two family groups separated in different tanks.

F2 parasites were selected for sensitivity towards H_2_O_2_ (Interox Paramove 50, H_2_O_2_ 50%, w/w; Solvay Chemicals, Brussels, Belgium) when they reached the adult stage. The selection was performed *in vitro* using two-dose bioassays at the Faculty of Veterinary Medicine, NMBU (University of Life Sciences, Oslo, Norway), starting within 6 h after sampling. All exposures were done in 1 l glass bottles held at 10–12 °C with constant aeration. The females were exposed to either 600 or 1800 ppm H_2_O_2_ for 30 min and the results were recorded immediately following exposure [11]. Control groups not exposed to H_2_O_2_ were included to check the general performance of the parasites. Parasites affected/immobilized at the lowest H_2_O_2_ concentration were considered sensitive, whereas parasites that were not visibly affected at the highest concentration were considered resistant. Lice were classified as affected when they were unable to attach to the container wall (lice could show weak swimming pattern, be partially or completely immobilized at the bottom of the container or float at the surface). Immediately after exposure and recording of the immobilization rate, lice were fixed in RNAlater and kept at − 80 °C following ~ 24 h at room temperature. Results were expressed as number and percentages of affected lice. A Chi-square test was used to test differences between family groups (statistically significance was assumed when *P* < 0.05). H_2_O_2_-sensitive and -resistant F2 adult females (named F2-S and F2-R, respectively) were used in the RNAseq analysis.

### Transcriptome analysis: samples and RNA extraction

In total, 36 adult female lice were enrolled in the transcriptome analysis. Details on their origin and group affiliation are given in Table 1. Total RNA was extracted from the individual adult females using a Trizol (Ambion, Life Technologies Thermo Fisher Scientific, Waltham, Massachusetts, USA) protocol combined with RNeasy Mini kit for animal tissues (Qiagen, Venlo, The Netherlands) (1 individual per extraction). Louse tissues were disrupted and homogenized in 1 ml Trizol using TissueLyser MM 301 (Qiagen Retsch, Venlo, The Netherlands) and one stainless steel bead of 5 mm diameter (Qiagen). After mixing with 0.2 ml of chloroform and a centrifugation step, the aqueous phase was transferred to a new vial and mixed with one volume of 70% ethanol. Total RNA was then isolated with RNeasy spin columns following the manufacturer’s protocol. Genomic DNA was removed from the extracted RNA (10 μg) with Turbo DNA-free kit (TURBO™ DNase Treatment and Removal Reagents; Ambion, Life Technologies Thermo Fisher Scientific). Subsequently, the RNA was cleaned and concentrated with RNA Clean & Concentrator™-5 kit (Zymo Research, CA, USA). The RNA was quantified with a ND-1000 Spectrophotometer (Thermo Fisher Scientific) and the quality was checked with a 2100 Bioanalyzer instrument (Agilent Technologies, CA, USA) and the Agilent RNA 6000 Nano kit.

**Table 1:** Data on the 36 samples used in the RNAseq study

Group	*n*	Description
Ls A-2013	4	Laboratory strain, sensitive to all delousing chemicals. Collected in northern Norway in 2011. Sixth generation. Not exposed to delousing chemicals during cultivation of any generation
Ls V-2013	5	Field strain, resistant to azamethiphos, deltamethrin, emamectin benzoate and hydrogen peroxide. Collected in mid-Norway in 2013
Ls A-P0	3	Laboratory strain. 12th generation of Ls A (sensitive). Not exposed to delousing chemicals during cultivation of any generation
Ls V-P0	4	Laboratory strain. Sixth generation of Ls V (resistant). Not exposed to delousing chemicals during cultivation of any generation
Ls F2-S	8	Second generation after crossing of Ls A-P0 and Ls V-P0, affected by 600 ppm H_2_O_2_ for 30 min (sensitive). Three lice from family group 1 and five lice from family group 2
Ls F2-R	12	Second generation after crossing of Ls A-P0 and Ls V-P0, unaffected by 1800 ppm H_2_O_2_ for 30 min (resistant). Seven lice from family group 1 and five lice from family group 2

*Notes*: All adult female lice. Family group 1: females from the sensitive Ls A strain were crossed with males from the H_2_O_2_-resistant Ls V strain in the P0 generation. Family group 2: males from the sensitive Ls A strain were crossed with females from the Ls V strain

*Abbreviation*: *n*, sample size

### Transcriptome analysis: RNAseq

Total RNA samples were used for library preparation and Illumina sequencing at the Norwegian Sequencing Centre (Oslo, Norway). Thirty-six RNAseq libraries (1 per individual louse), each with unique index barcodes, were prepared using the TruSeq Stranded total RNA library preparation Kit v2 (Illumina, CA, USA) following the manufacturer’s protocol including the polyA enrichment step. Libraries were pooled together and sequenced on NextSeq500 platform (Illumina) using 150 bp paired-end high-output reagents. Raw bcl files were generated using RTA v2.4.11 and were later demultiplexed (using the sample specific index) and converted to fastq format using bcl2fastq v2.17.1.14.

### Transcriptome analysis: gene expression analysis

Demultiplexed raw reads were cleaned using Trimmomatic v0.33 [20] to remove/trim low-quality reads and sequencing adapters as well as using BBMap v34.56 (https://sourceforge.net/projects/bbmap/) to remove reads mapping to the PhiX genome (Illumina spike-in). Cleaned fastq reads for each parasite were aligned to the *L. salmonis* transcriptome (coding sequences) using HiSat2 v2.1.0 [21]. The transcriptome file from ENSEMBL release 44 (ftp.ensemblgenomes.org/pub/metazoa/release-44/fasta/lepeophtheirus_salmonis) contained the predicted transcriptome from genomic data. It was modified for the aquaporin genes by substituting the predicted cds sequences in the original transcriptome with experimentally determined cds sequences from Stavang et al. [22]. Unmapped reads were filtered out using SAMtools v1.4 [23]. Gene annotation files in GTF format were generated for each parasite and then merged using Cufflinks v2.2.1. [24]. Counts of fragments aligning to each transcript were calculated using FeatureCounts v1.5.2. [25]. Analysis of the differential expression within each group (Ls A-2013 *vs* Ls V-2013; Ls A-P0 *vs* Ls V-P0; Ls F2-S *vs* Ls F2-R) were done using DESeq2 v 1.26.0 [26] (default settings for the count normalization method). The significance level was set to α = 0.05.

### Transcriptome analysis: differentially expressed genes shared between H_2_O_2_-resistant lice

The DESeq2 analysis generated two lists for each louse group (Ls 2013, Ls P0 and Ls F2), one list of genes upregulated in resistant lice and another list for genes downregulated in resistant lice, both compared to sensitive lice within the same group. Genes that were differentially expressed in the same direction in at least two of the three groups were identified. A Python-script (Additional file 1: Script S1 and Additional file 2: Script S2) was developed to identify the shared genes across all the groups or between two of the groups (Ls 2013 *vs* Ls P0, Ls 2013 *vs* Ls F2 and Ls P0 *vs* Ls F2). Genes were identified by their ENSEMBL name or the GenBank name [22]. The “ENSEMBL Metazoa (transcript)”, “protein information” section (http://metazoa.ensembl.org/Lepeophtheirus_salmonis/Info/Index), Uniprot database (https://www.uniprot.org/) and GenBank protein database (https://www.ncbi.nlm.nih.gov/genbank/) were used to annotate the genes shared across all the groups.

### H_2_O_2_ selection of Ls V lice

Lice from the H_2_O_2_-resistant strain (Ls V) kept in continuous laboratory culture without exposure to H_2_O_2_ for 4 years (2013–2017) were used as the first generation in an H_2_O_2_ selection experiment comprising 5 generations (F1-F5). Selection was performed on three generations (F1, F2 and F4) with 6 selection events: 3 on-fish and 3 off-fish. Selection on generation 3 (F3) could not be performed due to low lice numbers. The on- and off-fish selections allowed for two exposure events during the louse lifespan.

For the on-fish selection, fish infested with lice (mostly in the pre-adult I stage) were exposed to 1500 ppm H_2_O_2_ for 15–20 min (recommended concentration and exposure time for bath treatments) in a plastic container at 8.5–11 °C and under constant aeration. After treatment, fish were transferred to a recovery container with fresh seawater for 1 h, whereupon they were transferred back to their original tanks. Lice found in the treatment and recovery containers were discarded. The lice remaining on the fish were allowed to develop until females had reached the pre-adult II stage and then selected with H_2_O_2_ off-fish. The off-fish method allowed for selection of lice at higher H_2_O_2_ concentrations with good re-attachment to fish of the unaffected lice (90–100%). Briefly, lice were removed from anesthetized fish and transferred to 1 l glass bottles (25–50 lice per bottle), where they were exposed to 2000 or 2500 ppm H_2_O_2_ for 30 min. The water was gently mixed every 10 min. Exposures were performed within 4 h after sampling. Immediately after exposure, the condition of each louse was recorded. The bottles were emptied, and lice attached to the bottle walls were considered unaffected. The bottles were re-filled with 1 l of fresh seawater with constant aeration and lice were left for ~ 1 h. Unaffected parasites were then manually put back on the fish (5–10 males and 5–10 females per fish) by laying the lice on a plastic surface with their ventral side upwards, and slightly pressing and rolling one side of the anesthetized fish over all the lice that should infest that fish. Lice developed to adults and produced eggs for the next generation. Affected lice were discarded. Adult females could not be selected with H_2_O_2_ because they were not able to re-attach properly to fish after exposure.

Adult males and females from the F4 generation were removed from anesthetized fish and the egg strings were collected for hatching (F5). Adult F4 females were divided into two groups: one group was immediately fixed in RNAlater, and the other group was exposed to 1000 ppm H_2_O_2_ for 30 min (at 10 °C) prior to fixation in RNAlater. The sensitivity to H_2_O_2_ (EC_50_) was determined on the fifth generation (F5). Pre-adult II males and females, and young adult males were used to run a six-dose H_2_O_2_ bioassay in 2019. A six-dose exploratory H_2_O_2_ bioassay was performed before the selection as a reference in 2017. Both bioassay data were modelled using probit modelling in JMP software, and EC_50_ values with 90% confidence intervals were calculated separately for males and females. Generalized regression with binominal response distribution was used to test differences between before and after selection of the Ls V strain. Before or after selection and concentration, in addition to their interaction, were used as model effects. The test was run on males and females together as well as separated. Wald Chi-square (*χ*^2^), degrees of freedom (*df*) and the *P*-value are provided. Statistical significance was assumed when *P* < 0.05.

### qPCR study

Quantitative polymerase chain reaction (qPCR) was used to validate the RNAseq results for the unexposed adult females (Ls A-2013, Ls V-2013, Ls A-P0 and Ls V-P0) on two genes, *catalase* and *Glp1_v2*. An elevated expression of *catalase* has already been associated with resistance towards hydrogen peroxide in male and female pre-adult stages and in adult males [14], thus this gene was of special interest. The gene *Glp1_v2* was chosen since it was significantly downregulated in the three groups of H_2_O_2_-resistant parasites in the RNAseq study (Ls V-2013, Ls V-P0 and Ls F2-R), with a low adjusted *P*-value, *P*(adj), and relatively high log2 fold change. The two different quantification methods were compared for individual normalized counts (RNAseq) and ΔCq values (qPCR) for *catalase* and *Glp1_v2*. Correlation analysis were performed for each gene with Pearsonʼs correlation coefficient (linear fit) using JMP Pro 15.1.0 (SAS Institute Inc., 2019).

Two other qPCR analysis were performed to investigate the expression of *catalase* in two different sets of lice. One qPCR was run to test *catalase* expression in sensitive lice exposed to H_2_O_2_. Five adult females from the original laboratory Ls A strain were exposed to 600 ppm H_2_O_2_ for 30 min. Five unexposed lice were used as controls (calibrator sample). Only unaffected lice from both groups were included in the analysis. The other qPCR analysis tested the *catalase* expression on lice from the H_2_O_2_-selected Ls V strain (adult females of the F4 generation). Unexposed parasites (*n* = 5) or parasites exposed to 1000 ppm H_2_O_2_ for 30 min (*n* = 5; all unaffected after the exposure) were used. Ls V-P0 lice were included in the analysis to serve as controls before selection (calibrator sample).

RNA extraction, DNase treatment and RNA cleaning were performed for every sample the same way as samples prepared for RNAseq. First strand cDNA was produced from 1 µg of cleaned RNA using the qScript™ cDNA synthesis (reverse transcriptase) kit (Quanta Biosciences, MD, USA). The cDNA was cleaned with the DNA Clean & Concentrator™-5 kit (Zymo Research) and diluted 1:10 before being used as a PCR template for the qPCR using gene-specific primers and SsoAdvanced Universal SYBR Green Supermix (Bio-Rad, CA, USA), following the manufacturer’s protocol. Each qPCR reaction was optimized for 11 µl total reaction volume, 150/150 or 300/300 nM primer concentration and 2 µl of template, corresponding to 0.2 µg cDNA/RNA. Reactions were run in duplicate or triplicate and two negative controls were added, a non-template control and a no-reverse transcriptase control. The range of efficiencies for qPCR reactions were 96–98% for reference and target gene specific primers. The qPCR was run on a Bio-Rad CFX96 real-time system (Bio-Rad) under the following conditions: 95 °C for 30 s followed by 40 cycles of amplification at 95 °C for 10 s and 60 °C for 50 s. After qPCR, the homogeneity and specificity of the PCR products was confirmed by melting curve analysis, agarose gel electrophoresis and Sanger sequencing. Relative gene expression was determined by the ΔCq method (ΔCq = Cq_target_ − Cq_reference_), where Cq_target_ is the Cq values for *catalase* or *Glp1_v2*, and Cq_reference_ the average of the elongation factor 1-alpha and prohibitin-2 genes (see Table 2 for primer details). The use of two reference genes with different expression levels (high for elongation factor 1-alpha and low for prohibitin-2, in adult female lice) is beneficial for the qPCR accuracy when the expression of the target genes in the different samples is expected to have a relatively big range. The expression of both reference genes was stable under H_2_O_2_ exposure in adult *L. salmonis* females. Fold change in gene expression was calculated according to the 2^−(∆∆Cq)^ method, using the Cq values of the corresponding control groups as calibrator sample.

**Table 2:** Primers used in the qPCR study

Gene	Primer name	Primer sequence	Primer concentration (nM)	Product size (bp)
*Catalase*	Ls_Cat_6 F	CCACAGAACAACTTGCCAAC	150/150	157
	Ls_Cat_6 R	GCCATTTCGTCCATAAATGC		
*Glp1_v2*	Ls_Glp1_2 F	TCGGCTCCAGGAATTGTTCT	300/300	200
	Ls_Glp1_2 R	GGTCCTAAATCTCTCGCTGGG		
*Elongation factor 1-alpha*	Ls_gEF_2 F	ATGGCACGGAGACAACATGT	150/150	206
	Ls_gEF_2 R	CGGGCACTGTTCCAATACCT		
*Prohibitin-2*	Ls_gProhib2_2 F	GCTCATCACACAGCGTCAAC	300/300	176
	Ls_gProhib2_2 R	CAGCTCTTTGGGCCTCTTGT		

## Results and discussion

### Crossing experiment and bioassays

In order to obtain both H_2_O_2_-sensitive and H_2_O_2_-resistant lice for the RNAseq study, F2 adult females were selected with two-dose H_2_O_2_ bioassays. F2 lice belonged to 2 different families, originating from batch crossing of sensitive (Ls A) and resistant (Ls V) lice. Table 3 shows the number of F2 adult females affected at the different H_2_O_2_ doses for each family group. There were no significant differences between the family groups (*χ*^2^ = 0.023, *df* = 1, *P* = 0.88), indicating that inheritance of resistance was not sex-specific (i.e. there were similar numbers of affected lice independently of which strain the P0 males and females belonged to).

**Table 3:** Number of F2 adult female lice affected in two-dose H_2_O_2_ bioassays

	Crossing and bioassays		
	Family group 1	Family group 1	Family group 2
0 ppm (Control)	1/18 (6%)	0/5 (0%)	1/18 (6%)
600 ppm	2/16 (13%)	1/18 (6%)	8/32 (25%)
1800 ppm	13/15 (87%)	12/18 (67%)	16/25 (64%)

*Notes*: Bioassays: 30 min exposure; three bioassays in total (two using lice from family group 1 and one with lice from family group 2). Results indicated as fractions (number of affected lice out of total lice per dose) and percentages (in parentheses). Family group 1, females from the sensitive Ls A strain were crossed with males from the H_2_O_2_-resistant Ls V strain in the P0 generation; family group 2, males from the sensitive Ls A strain were crossed with females from the Ls V strain

### RNAseq expression analysis

**Fig. 1:**
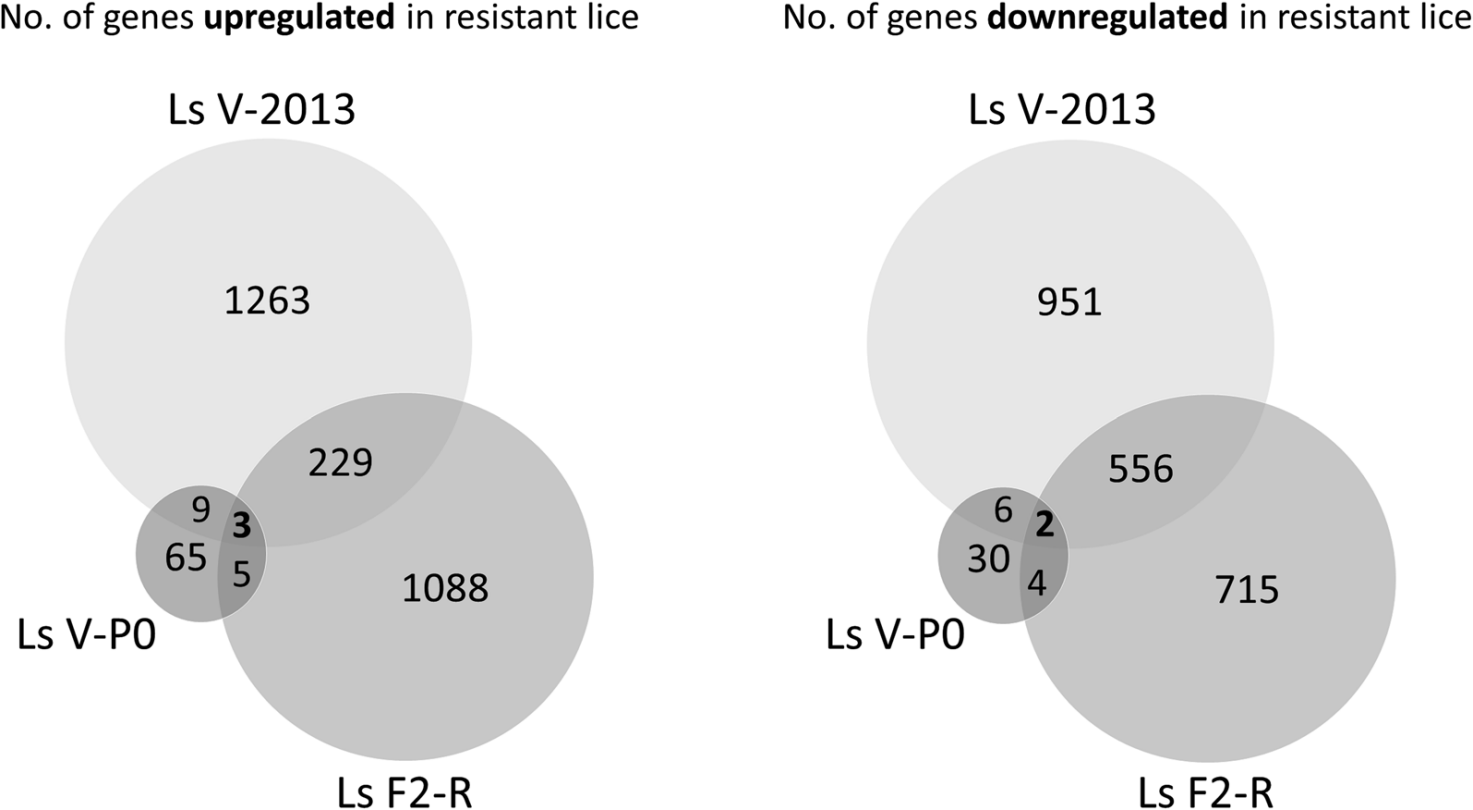
Number of genes differentially expressed in the H_2_O_2_-resistant lice groups (Ls V-2013, Ls V-P0 and Ls F2-R) *versus* the corresponding sensitive groups (Ls A-2013, Ls A-P0 and Ls F2-S), separately for up- and downregulated genes. Numbers in the circles but outside the intersections represent the genes differentially expressed in only one group. Numbers in the intersection of the circles represent the differentially expressed genes shared between two or three groups

RNAseq gene expression analysis (DESeq2) showed that the groups Ls V-2013 and Ls F2-R each had more than 2000 genes differentially regulated compared to the corresponding sensitive groups, Ls A-2013 and Ls F2-S (Fig. 1, Additional file 3: Dataset S1). The Ls V-P0 lice had less than 150 genes differentially regulated compared to Ls A-P0.

The number of differentially expressed genes in the original resistant and sensitive strains collected in 2013 (see Table 1 for strain details), likely reflect both genes associated with resistance and genes necessary for adaptation to different environments. In the P0 generation, environmental conditions had been similar between the strains for two years and without any exposure to medicinal compounds, thus some differences related to environmental adaptation were likely evened out. The subsequent increase of differentially expressed genes from P0 generations to the H_2_O_2_-selected F2-generation could suggest induction of a high number of genes due to the H_2_O_2_ exposure. The F2 parasites were sampled immediately after a 30 min H_2_O_2_ exposure, thus differential regulation of the expression of a number of genes as a consequence of this exposure within this short time frame could be questioned. It has though been demonstrated that induction of genes needed to cope with oxidative stress can happen within two hours [27], possibly also sooner, although this has not been studied. Several putative methyltransferases and transcription factors (typically involved in gene transcription activation or repression) were found differentially expressed in our RNAseq study (data not shown), supporting the induction theory.

### *Catalase* expression

**Fig. 2:**
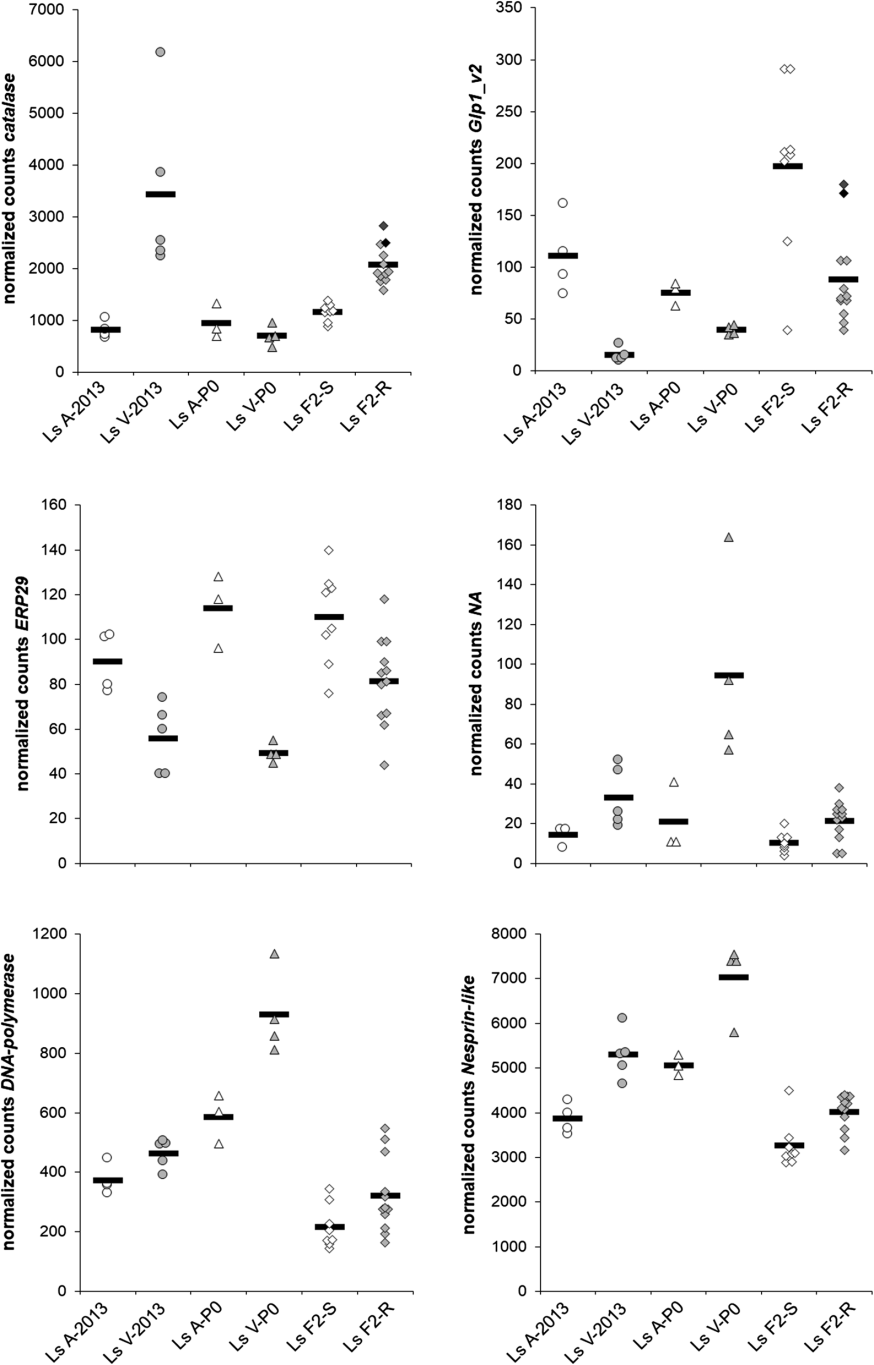
Gene expression data (normalized counts from the RNAseq study) of *catalase* and five genes significantly differentially expressed across 2013, P0 and F2 groups (*DNA-polymerase*, *Nesprin-like*, *NA*, *ERP29* and *Glp1_v2*). Ls A-2013 (white circles), Ls V-2013 (grey circles), Ls A-P0 (white triangles), Ls V-P0 (grey triangles), Ls F2-S (white diamonds), Ls F2-R (grey diamonds). Ls A/F2-S represent the sensitive lice, and Ls V/F2-R, the resistant ones. Solid lines represent the arithmetic mean in each group. Dark grey and black diamonds in the Ls F2-R group correspond to the same individual lice in both *catalase* and *Glp1_v2* graphs

**Fig. 3:**
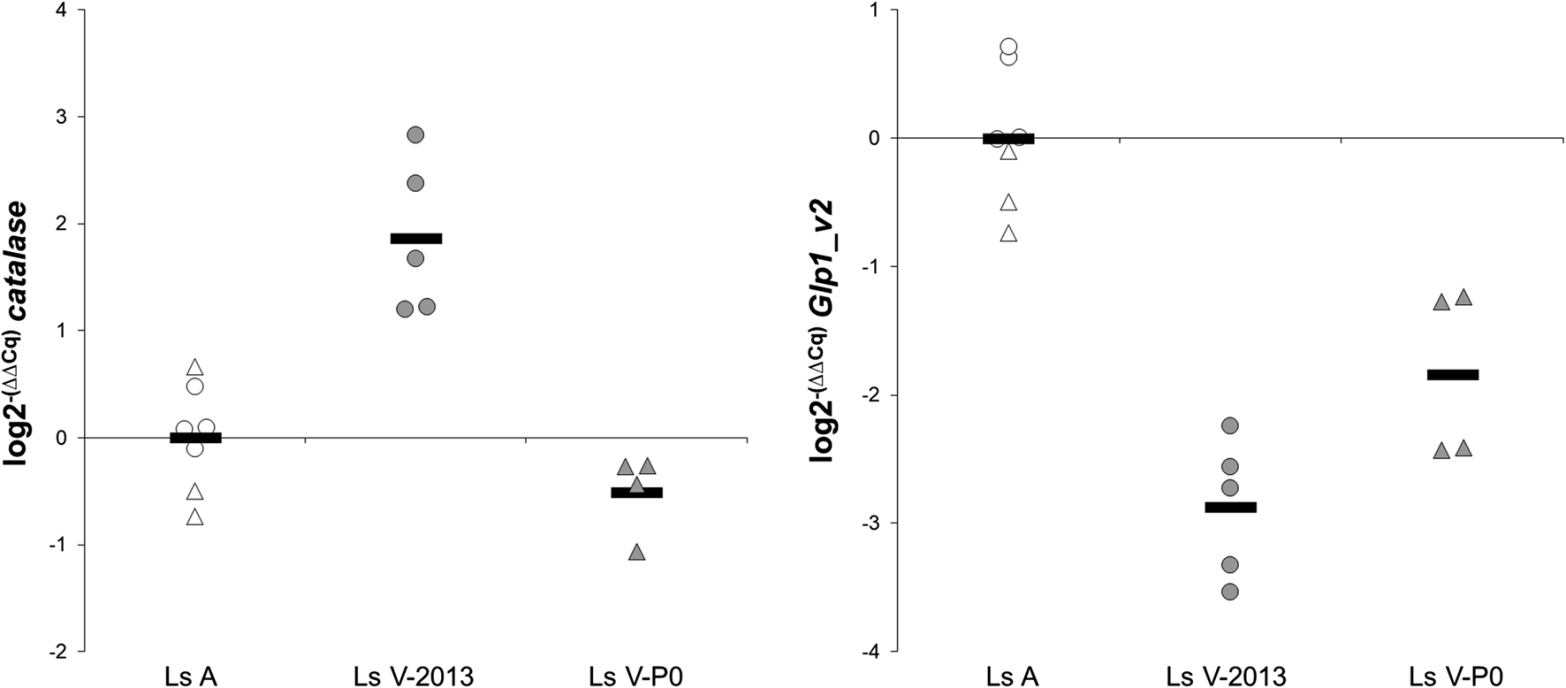
qPCR validation study for *catalase* and *Glp1_v2* genes in the louse groups Ls A-2013 (white circles), Ls V-2013 (grey circles), Ls A-P0 (white triangles) and Ls V-P0 (grey triangles). Ls A represent the sensitive lice, and Ls V, the resistant lice. Solid lines represent the arithmetic mean in each group. Data shown as fold change (log2^−(∆∆Cq)^) referred to Ls A (Ls A-2013 and Ls A-P0) (calibrator sample). Statistical analysis was not performed due to the low sample size in some of the groups

**Fig. 4:**
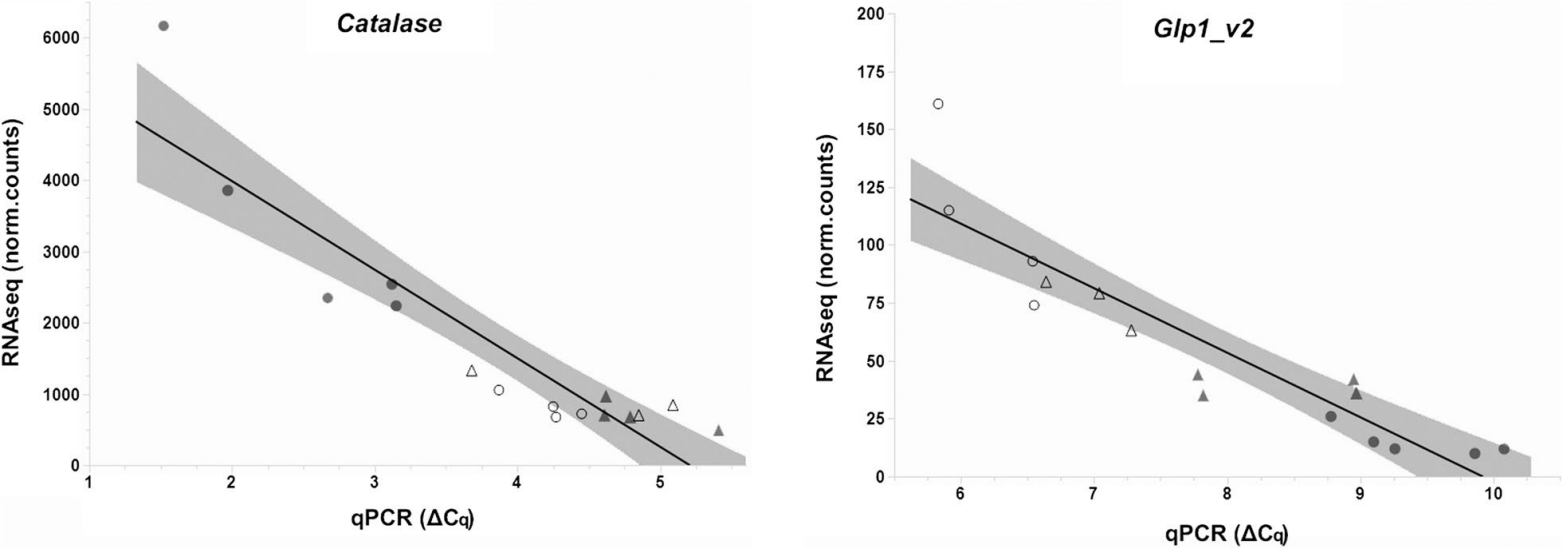
Correlation between RNAseq (normalized counts) and qPCR (ΔCq values) for the expression of *catalase* and *Glp1_v2* in sensitive (Ls A-2013 and Ls A-P0; white circles and triangles, respectively) and resistant lice (Ls V-2013 and Ls V-P0; grey circles and triangles, respectively). A linear fit with the 95% confidence interval (shaded area) has been added. Pearsonʼs correlation coefficient (*r*) was calculated to test the strength of the linear fit (statistically significant if *P* < 0.05)

The *catalase* gene was previously found differentially expressed in H_2_O_2_-sensitive and -resistant lice [14] and its expression level has been introduced as a H_2_O_2_ resistance marker in the salmon industry [15]. The present RNAseq study sought to validate the use of *catalase* expression as a resistance marker in adult females, as this developmental stage was not included in a previous study [14]. There were significantly higher numbers of *catalase* transcripts in resistant lice exposed to H_2_O_2_ (Ls V-2013 and Ls F2-R) than in sensitive lice (Table 4, Fig. 2). However, the number of *catalase* transcripts in the P0 generation of Ls V, a H_2_O_2_-resistant strain that was unexposed to H_2_O_2_ for several generations, did not differ significantly from the sensitive Ls A-P0. The qPCR validation confirmed the gene expression pattern found for 2013 and P0 RNAseq samples: Ls V-2013 had higher *catalase* expression than Ls A, while the expression in Ls V-P0 and Ls A was similar (Figs. 2, 3 and 4).

**Table 4:** Gene expression data of several genes differentially expressed in the louse groups Ls 2013, P0 and F2

Gene	Lice group	Normalized counts: arithmetic mean ± SD (range)		log2FC	*P*(adj)
		Ls A/F2-S	Ls V/F2-R		
*Catalase*	2013	819 ± 169 (675–1055)	3429 ± 1662 (2236–6165)	2.07	**< 0.001**
	P0	954 ± 330 (696–1326)	706 ± 195 (491–963)	− 0.43	0.784
	F2	1161 ± 164 (891–1386)	2072 ± 366 (1580–2821)	0.84	**< 0.001**
*DNA-polymerase*	2013	374 ± 50 (331–447)	464 ± 49 (390–505)	0.32	**0.044**
	P0	585 ± 83 (495–658)	930 ± 142 (812–1134)	0.67	**0.024**
	F2	217 ± 73 (144–344)	320 ± 125 (165–548)	0.56	**0.045**
*Nesprin-like*	2013	3865 ± 345 (3522–4290)	5297 ± 538 (4644–6116)	0.46	**< 0.001**
	P0	5066 ± 234 (4837–5304)	7036 ± 825 (5803–7547)	0.47	**0.034**
	F2	3271 ± 527 (2887–4498)	4021 ± 409 (3158–4403)	0.30	**0.005**
*NA*	2013	14 ± 4 (8–17)	33 ± 15 (19–52)	1.19	**0.018**
	P0	21 ± 17 (11–41)	95 ± 49 (57–164)	2.16	**0.026**
	F2	10 ± 5 (4–20)	21 ± 10 (5–38)	1.03	**0.015**
*ERP29*	2013	90 ± 13 (77–102)	56 ± 15 (40–74)	− 0.69	**0.015**
	P0	114 ± 16 (96–128)	50 ± 4 (45–55)	− 1.20	**< 0.001**
	F2	110 ± 21 (76–140)	81 ± 20 (44–118)	− 0.44	**0.019**
*Glp1_v2*	2013	112 ± 39 (74–164)	15 ± 6 (10–26)	− 2.89	**< 0.001**
	P0	77 ± 11 (64–86)	40 ± 5 (35–44)	− 0.93	**0.025**
	F2	197 ± 83 (39–292)	88 ± 45 (40–181)	− 1.16	**0.002**
*Aqp12L1*	2013	158 ± 14 (140–173)	99 ± 32 (73–152)	− 0.68	**0.013**
	P0	162 ± 26 (144–192)	148 ± 23 (130–181)	− 0.13	0.957
	F2	182 ± 29 (130–219)	141 ± 25 (104–185)	− 0.37	**0.010**
*Aqp12L2*	2013	56 ± 14 (42–75)	15 ± 3 (13–20)	− 1.91	**< 0.001**
	P0	29 ± 18 (11–46)	24 ± 5 (19–30)	− 0.29	0.960
	F2	98 ± 18 (69–124)	66 ± 21 (31–103)	− 0.57	**0.012**
*Glp2*	2013	21 ± 7 (15–31)	5 ± 6 (0–14)	− 1.98	**0.045**
	P0	17 ± 10 (7–26)	20 ± 7 (16–30)	0.22	0.976
	F2	25 ± 12 (6–42)	11 ± 5 (1–17)	− 1.12	**0.008**
*Glp3_v1*	2013	149 ± 30 (110–182)	297 ± 74 (183–365)	0.99	**< 0.001**
	P0	222 ± 34 (185–253)	174 ± 56 (120–253)	− 0.35	0.855
	F2	134 ± 34 (91–203)	121 ± 18 (82–148)	− 0.16	0.378

Number of lice included in each group (*n*) is provided in Table 1

*Notes*: Upregulation is indicated as log2FC positive values; downregulation as log2FC negative values. Statistical significance is indicated in bold (*P*(adj) values). ENSEMBL *L. salmonis* transcriptome was used in the analysis, but the sequences of genes coding for aquaporins were replaced by GenBank entries: *Catalase*, EMLSAT00000007315; *DNA-polymerase*, EMLSAT00000002584; *Nesprin-like*, EMLSAT00000005972; *NA* (unannotated), EMLSAT00000005947; *ERP29*, EMLSAT00000009549; *Glp1_v2*, KR005661.1; *Aqp12L1*, KR005665.1; *Aqp12L2*, KR005666.1; *Glp2*, KR005662.1; *Glp3_v1*, KR005663.1

*Abbreviations*: Ls A/F2-S, sensitive lice; Ls V/F2-R, resistant lice; SD, standard deviation; Log2FC, log2 fold change; *P*(adj), *P*-value for normalized counts (α = 0.05)

Two six-dose H_2_O_2_ bioassays were run after completion of the RNAseq study to check the sensitivity of the unexposed Ls V strain. The EC_50_ value for pre-adult II females from the Ls V laboratory strain was 1635 ppm, eight times higher than the Ls A strain (216 ppm) (Table 5; 2017 bioassay for Ls V), and the value for Ls V adult females was 1063 ppm (90% CI: 664–1703; *n* = 34), suggesting that Ls V-P0 lice were still resistant to H_2_O_2_ when enrolled in the RNAseq study. Based on the results from the two-dose H_2_O_2_ bioassays performed on F2 lice, the Ls V-P0 descendants, the EC_50_ value for F2 lice would be expected between 600 and 1800 ppm (Table 3), almost three times higher than the value for Ls A lice. DESeq2 analysis for Ls F2-R showed that these lice had on average close to three times higher numbers of *catalase* transcripts than their grandparents, Ls V-P0 (Table 4). In addition, *catalase* was one of the most important differentially expressed genes in Ls F2-R lice, having the lowest *P*(adj) value and without overlap in the range of normalized counts between F2 sensitive and resistant lice, efficiently separating those groups. These results indicate that *catalase* expression is induced by H_2_O_2_ exposure in resistant lice. The induction of *catalase* expression after H_2_O_2_ exposure has previously been demonstrated in a penaeid shrimp. The gene was significantly upregulated 2 h after injecting 0.1% H_2_O_2_ in the shrimp body [27].

**Table 5:** Bioassay data for pre-adult II (males and females) and young adult males exposed to H_2_O_2_ for 30 min

Louse strain	H_2_O_2_ exposure data	EC_50_ (ppm) (90% CI)
Ls A laboratory strain (Ref)^a^	2013; 10–12 °C; *N* and dose	216 (153–305)
Ls V F0 (Ref)^a^	2013; 10–12 °C; *N* and dose	2127 (1253–3610)
Ls V F1 (Ref)^a^	2013; 10–12 °C; *N* and dose	1767 (1494–2090)
Ls V laboratory strain before H_2_O_2_ selection	2017; 10–11 °C; *N*: 25 females and 22 males; 0, 600, 1400, 2200, 3000, 4200 ppm	Females: 1635 (734–3643); Males: 1795 (1095–2943)
Ls V laboratory strain (F5) after H_2_O_2_ selection	2019; 10 °C; *N*: 130 females and 118 males; 0, 600, 1400, 2200, 3000, 4200 ppm	Females: 2441 (2012–2961); Males: 1861 (1482–2337)

*Notes*: H_2_O_2_ exposure data: year; water temperature; *N*, total number of lice used (all chemical concentrations together); doses (ppm = mg l^−1^)

^a^Ref: Previously published data for Ls V (resistant strain) and Ls A (sensitive strain) in Helgesen et al. 2015 [11]. EC_50_ values for males and females together; 95% CI. General nominal concentration ranging from 0 to 5000 ppm, adjusted for each strain. N not available

*Abbreviations*: EC_50_, concentration affecting 50% of the lice; CI, confidence interval; Ls A, sensitive strain; Ls V, H_2_O_2_-resistant strain

**Fig. 5:**
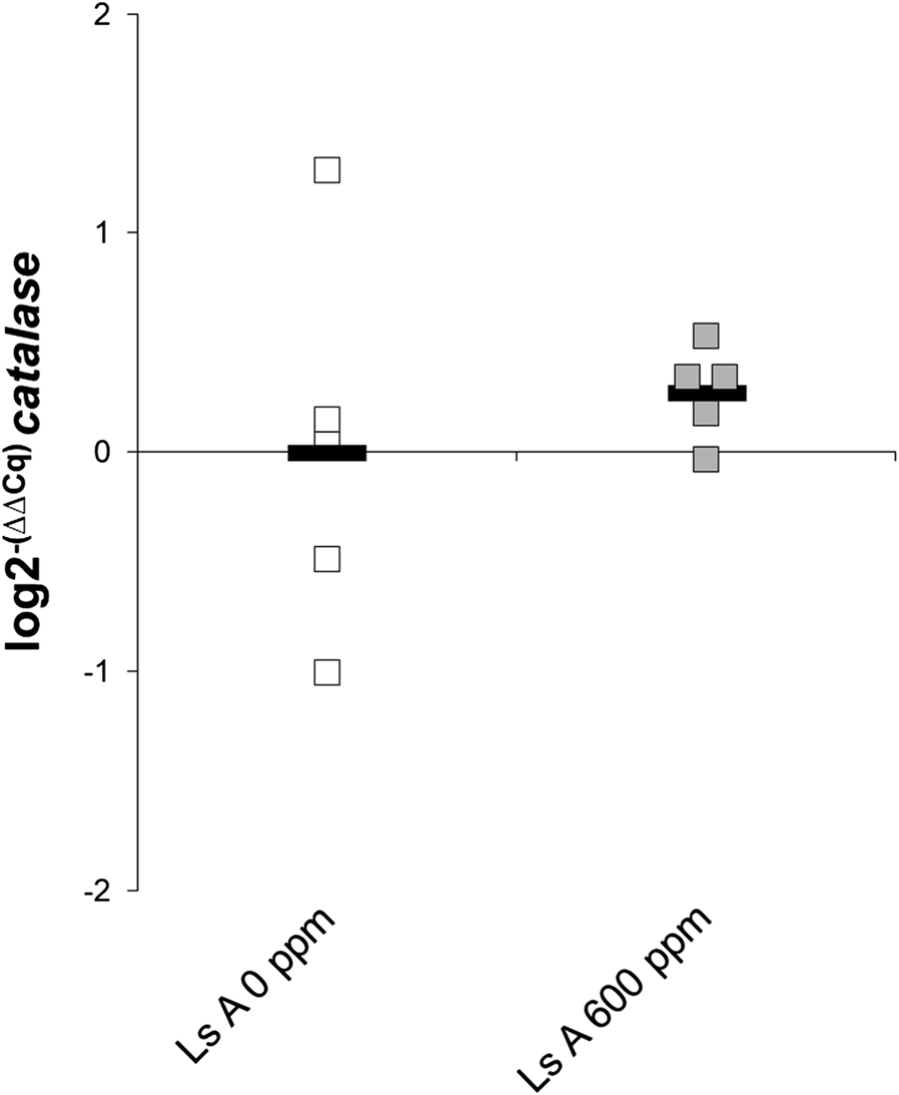
qPCR study for *catalase* expression in the original laboratory Ls A strain (sensitive to H_2_O_2_, adult females). Ls A 0 ppm: unexposed lice (*n* = 5; white rectangles); Ls A 600 ppm: lice exposed to 600 ppm H_2_O_2_ for 30 min (*n* = 5; grey rectangles; unaffected after the exposure). Solid lines represent the arithmetic mean in each group. Data shown as fold change (log2^−(∆∆Cq)^) referred to Ls A 0 ppm lice (calibrator sample). Statistical analysis was not performed due to the low sample size in each group

In contrast to resistant lice, sensitive lice do not appear to induce *catalase* expression following H_2_O_2_ exposure. This was shown with qPCR on adult females from the sensitive Ls A strain unaffected after 30 min exposure to 600 ppm H_2_O_2_, when compared to a parallel group of unexposed Ls A females (Fig. 5). However, the inter-individual variation in the exposed group, was smaller than in the unexposed one. A similar trend is observed among sensitive lice enrolled in the RNAseq study: Ls A-2013 and Ls A-P0 (unexposed) *vs* F2-S (exposed) (Table 4, Fig. 2).

H_2_O_2_ resistance has been demonstrated to be hereditary [11, 14]. The heritable factor may thus be the ability to quickly induce *catalase* expression. The induction of *catalase* expression poses a challenge for its use as an H_2_O_2_ resistance marker, since unexposed resistant lice may have low *catalase* expression and could erroneously be classified as sensitive. On the other hand, after a short exposure to H_2_O_2_, sensitive and resistant lice seem to be easily separable by *catalase* expression.

### New candidate genes

To identify more genes associated with H_2_O_2_ resistance, differentially expressed genes from the Ls 2013, Ls P0 and Ls F2 generations were compared. The resistant lice that had been exposed to H_2_O_2_ (Ls V-2013 and Ls F2-R) shared 790 differentially expressed genes (Fig. 1). This supports the hypothesis that H_2_O_2_ exposure can induce the expression of several genes, even within a time-span of 30 min. The complete list of genes shared across two or all three groups is presented in Additional file 4: Dataset S2.

Only five genes (three upregulated and two downregulated in resistant lice) were differentially expressed in all three groups (Ls V-2013, Ls V-P0 and Ls F2-R) (Figs. 1, 2), thus irrespective of H_2_O_2_ exposure. Table 4 shows the gene expression and annotation data for those genes. The fold change ranged from *c.*1.2 to 8, up- or downregulated depending on the gene. The three genes consistently upregulated in resistant lice encoded a DNA polymerase (delta subunit 3), a Nesprin-like protein and an unannotated small protein (named NA; 77 aa long). DNA polymerase is an enzyme that synthesize DNA from deoxyribonucleotides, and the delta subunit 3 plays a role in high fidelity genome replication. The protein identified as Nesprin-like contained a KASH domain and a spectrin repeat (spectrin/alpha-actinin). It probably belongs to the Nesprin-1 or -2 type, actin-binding proteins involved in the maintenance of nuclear organization and structural integrity. The NA protein might be a mini-protein with regulatory functions. A large amount of mini- and micro-proteins (small proteins usually < 100 aa long) acting as negative or positive regulators, have been identified in unicellular organisms, plants and animals [28, 29]. For example, some small proteins sequester their targets into non-functional complexes, others attract chromatin repressor proteins, or others interact with ion channels compromising their transport capacity.

The two genes downregulated in all resistant lice were the genes encoding endoplasmic reticulum resident protein 29 (*ERP29*) and an aquaporin protein (*Glp1_v2*). ERP29 plays an important role in the processing of secretory proteins within the endoplasmic reticulum. Aquaporins are protein channels that facilitate the rapid transport of water and other small solutes such as H_2_O_2_ and gasses [22, 30–34].

The most interesting, differentially expressed gene was *Glp1_v2*, one of the aquaglyceroporins (Glp) identified by Stavang et al. [22] in *L. salmonis*. Stavang et al. [22] identified a total of seven aquaporins, with several splice variants, in the salmon louse: two classical aquaporins, Bib and PripL (Prip-like); three aquaglyceroporins, Glp1_v1, Glp1_v2, Glp2, Glp3_v1 and Glp3_v2 (v1 and v2 represent the splice variants); and two unorthodox aquaporins, Aqp12L1 (Aqp12-like1) and Aqp12L2 (Aqp12-like 2). All but *Glp1_v1* and *Glp3_v2* were detected in our RNAseq data. Stavang et al. [22] reported *Glp1_v1* only in pre-adult II and adult males, while *Glp1_v2* was detected in both sexes. *Glp3_v2* was expressed mostly in nauplius II stage. Table 4 shows the gene expression data for several aquaporins in our study. There were no statistically significant differences in the expression of *Bib* or *PripL* within any of the Ls 2013, Ls P0 or Ls F2 groups (data not shown). However, *Glp1_v2* was statistically significantly downregulated in all H_2_O_2_-resistant groups (Ls V-2013, Ls V-P0 and Ls F2-R). The qPCR analysis revealed a similar gene expression pattern, with Ls V-2013 and Ls V-P0 having lower *Glp1_v2* expression levels compared to the corresponding Ls A groups (Figs. 2, 3 and 4). *Glp 2* was significantly downregulated in two groups, Ls V-2013 and Ls F2-R, but the expression of this gene was low. *Glp3_v1* was upregulated in only Ls V-2013. The unorthodox aquaporins, *Aqp12L1* and *Aqp12L2*, were statistically significantly downregulated in Ls V-2013 and Ls F2-R groups, but not in the Ls V-P0 lice.

It has been demonstrated that certain aquaglyceroporins and unorthodox aquaporins are able to transport H_2_O_2_ through cell membranes in several organisms [32, 33]. Glps have an open pore configuration in *L. salmonis* [22], allowing bigger molecules than water, like urea and glycerol, to pass through the channel. Miller et al. [32], found that one aquaglyceroporin (AQP3) and one unorthodox aquaporin (AQP8) transported H_2_O_2_ through mammalian cell membranes. However, the classical aquaporin AQP1, did not transport H_2_O_2_. As *Glp1_v2* was downregulated in all three groups of H_2_O_2_-resistant lice in the current study, a possible involvement in the influx or distribution of H_2_O_2_ in the salmon louse body or cells seems probable; the lower the number of Glp1_v2 channels, the lesser amount of exogenous H_2_O_2_ can enter and cause toxic effects. The downregulation of *Aqp12L1* and *Aqp12L2* in resistant lice exposed to H_2_O_2_ may also indicate a role of these proteins as H_2_O_2_ channels. This goes especially for *Aqp12L2*, with almost 4-fold downregulation and very low *P*(adj) value (< 0.001) in the Ls 2013 groups (Table 4). As in the case of Glps, Stavang et al. [22] also found an open pore configuration in the 3D modelling of Aqp12L2.

Several authors have reported the role of aquaporins as drug transporters in other parasites, as well as a link between aquaporins and drug resistance [35]. Faghiri & Skelly [36], showed the presence of a putative aquaglyceroporin (SmAQP) in the tegument of the parasitic worm *Schistosoma mansoni*. It was proven that SmAQP can transport water and an anti-parasitic compound (potassium antimonyl tartrate) across the parasite tegument. In addition, parasites with reduced levels of SmAQP exhibited a greater resistance to the anti-parasitic agent. In trypanosomatid parasites, such as *Leishmania* spp. and *Trypanosoma* spp., certain aquaporins transport trivalent metalloids (SbIII and AsIII) through the parasite membranes [37]. The aquaglyceroporin LmAQP1 transports SbIII in *Leishmania* spp. [38]. Drug-resistant parasites showed downregulation of the *LmAQP1* gene [39], and RNA levels correlated with drug concentration. Resistance to melarsoprol and pentamidine is common among African trypanosomes [40]. The authors found that the loss of function of an aquaglyceroporin, AQP2, was linked to drug resistance.

Studies have shown that the amount of functional proteins can be related to the amount of RNA transcripts, but also to the activation state or degradation rate of the proteins. For example, a mitogen-activated protein kinase 2 (MPK2) stabilizes LmAQP1 protein by phosphorylation in *Leishmania major* [41], and dephosphorylation made LmAQP1 more susceptible to degradation. Altered AQP1 and MPK2 (by site-directed mutagenesis) reduced the drug uptake and drug sensitivity. Catalase activity can also be regulated by reversible phosphorylation *via* kinase enzymes by increasing the affinity of the enzyme for H_2_O_2_ [42]. In our *L. salmonis* RNAseq study, we found four putative mitogen-activated protein kinases differentially expressed in H_2_O_2_-sensitive and H_2_O_2_-resistant lice (data not shown), indicating that drug sensitivity might be linked to regulation of gene expression, but also to the amount and functionality of the proteins.

The role of *DNA-polymerase*, *Nesprin-like*, *NA* and *ERP29* in H_2_O_2_ resistance is difficult to establish. Nevertheless, these genes, together with *Glp1_v2*, may become very interesting candidate genes for developing molecular markers for monitoring H_2_O_2_ resistance, since they are consistently up- or downregulated in all resistant louse groups.

Only one H_2_O_2_-resistant strain (Ls V) could be included in the present study, which make generalizations about the H_2_O_2_ resistance mechanisms and markers challenging. However, F2 resistant lice might be considered a different lice population/strain since it was a mix of a sensitive (Ls A) and a resistant strain (Ls V). F2 lice had a wide range of H_2_O_2_ sensitivities, with some individuals affected at 600 ppm H_2_O_2_ and some unaffected at 1800 ppm. At the molecular level, there were statistically significant differences between sensitive and resistant F2 lice in normalized counts for *DNA-polymerase*, *Nesprin-like*, *NA*, *ERP29* and *Glp1_v2*, but there were overlaps in the group ranges for all of these genes (Table 4). This overlap may suggest that H_2_O_2_ resistance in F2 lice came from several up- and downregulated genes combined in slightly different ways, enabling individual parasites to survive 1800 ppm H_2_O_2_. As an example, the two F2 resistant lice with high number of *Glp1_v2* reads (Fig. 2, dark grey and black diamonds), are the ones with higher *catalase* expression, possibly suggesting a compensatory effect: high numbers of Glp1_v2 could mean that more exogenous H_2_O_2_ would enter the louse body and cells. The louse would then need more catalase for breaking down the H_2_O_2_ and survive the exposure. This observation on the expression overlap of several genes, suggests that the H_2_O_2_-resistance mechanisms can vary slightly between individuals. However, the general resistance pattern is the same, at least in related louse strains. According to these observations, we propose the development of a set of molecular markers based on the expression of *catalase*, *Glp1_v2*, *DNA-polymerase*, *Nesprin-like*, *NA* and *ERP29* genes, that should be validated on other louse populations/strains.

The use of gene expression as molecular marker for reduced sensitivity towards a treatment is challenging, since the expression may also be affected by other factors. For example, different dosages, exposure times, temperature, handling stress, developmental stage and sex may all influence gene expression, regardless of treatment tolerance. A standardized protocol for lice collection, handling, fixation and H_2_O_2_ exposure (if necessary), is thus warranted for an efficient performance of gene expression markers. A combination of the expression levels of several genes may provide a more robust tool, as different genes may be sensitive to different factors.

### Correlation between RNAseq and qPCR results

The Ls A and Ls V lice from 2013 and the P0 generation were subjected to both RNAseq and qPCR analysis for the expression of *catalase* and *Glp1_v2*. The strength of the linear relationship (Pearsonʼs correlation coefficient, *r*) between the two measurements were calculated to be *r*_(15)_ = − 0.917, *P* < 0.0001 and *r*_(15)_ = − 0.916, *P* < 0.0001, for catalase and Glp1_v2, respectively (Fig. 4). Both RNAseq analysis and qPCR analysis separated the groups similarly (Figs. 2, 3).

### H_2_O_2_ selection of resistant lice

The H_2_O_2_-resistant strain (Ls V) was followed for five generations, of which three were H_2_O_2_-selected. The aims were to study if the resistant Ls V laboratory strain could further increase its resistance level and to study *catalase* expression after selection. Table 6 shows the percentage of affected lice after each H_2_O_2_ exposure. The H_2_O_2_ sensitivity was tested before selection and on the fifth generation (F5) of selected lice (Table 5). The EC_50_ for F5 males was similar to the value before selection (*χ*^2^ = 1.27, *df* = 1, *P* = 0.259). The EC_50_ value for females increased slightly after selection (*c.*1.5 times), although this difference was not statistically significant (*χ*^2^ = 2.11, *df* = 1, *P* = 0.147). At the population level, the resistant Ls V laboratory strain may have increased its resistance only to a level similar to the field lice that originated the laboratory strain, which were exposed to H_2_O_2_ several times in the field (Table 5, Ls V F0). Thus, it could be possible that Ls V had reached its maximum level of tolerance.

**Table 6:** H_2_O_2_ selection experiment of the H_2_O_2_-resistant strain Ls V: design and results (% affected lice)

F	H_2_O_2_ exposure data	Louse instar (*n*)	% affected lice
F1	FBT (4): 1500 ppm, 15–20 min, 11 °C	Pre-adult I-II (250)	8
	BIO: 2000 ppm, 30 min, 11 °C	Pre-adult II – adult males (180)	33
F2	FBT (4): 1500 ppm, 15–20 min, 8.5 °C	Pre-adult I-II (150)	7
	BIO: 2500 ppm, 30 min, 8.5 °C	Pre-adult II – adult males (110)	50
F3	Not selected with H_2_O_2_	–	–
F4	FBT (16): 1500 ppm, 15–20 min, 8.5 °C	Pre-adult I-II (360)	8.3
	BIO: 2500 ppm, 30 min, 11 °C	Pre-adult II – adult males (312)	63
F5	Six-dose bioassay	Pre-adult II – adult males	–

*Notes*: F: lice generation (this F2 generation is not the same as the F2 generation from the crossing and RNAseq experiments). H_2_O_2_ exposure type and data: FBT (fish bath treatment), lice treated on-fish using a bath treatment methodology (number of fish used in parentheses); BIO (bioassay selection), lice treated off-fish using a bioassay methodology; H_2_O_2_ concentration, exposure time, water temperature. Instar: louse developmental stage; *n*: number (approximately) of lice used in each selection event (males and females together) in parentheses. Selection on generation 3 (F3) could not be performed due to low lice numbers. F5 was not selected with H_2_O_2_; this generation was used to test the H_2_O_2_ sensitivity after selection using a six-dose bioassay (see bioassay details in Table 5)

**Fig. 6:**
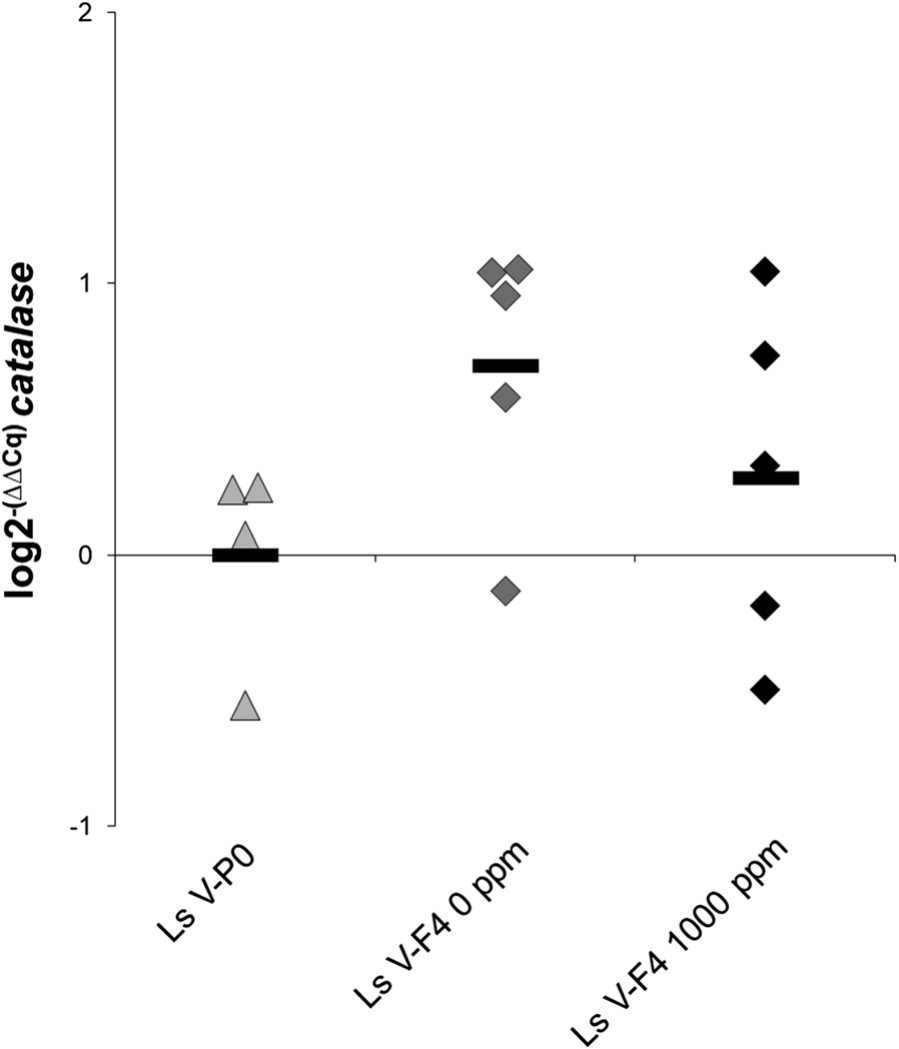
qPCR study for *catalase* expression in H_2_O_2_-selected Ls V lice (F4 generation; adult females) and in Ls V-P0 lice from the RNAseq study: Ls V-P0 (grey triangles). Ls V-F4 0 ppm: selected Ls V lice not exposed to H_2_O_2_ before fixation (dark grey diamonds); Ls V-F4 1000 ppm: selected Ls V lice exposed to 1000 ppm H_2_O_2_ for 30 min immediately before fixation (black diamonds). Solid lines represent the arithmetic mean in each group. Data shown as fold change (log2^−(∆∆Cq)^) referred to Ls V-P0 lice (calibrator sample). Statistical analysis was not performed due to the low sample size in each group

*Catalase* expression was investigated in the H_2_O_2_-selected branch of the Ls V strain (F4 generation) and compared with Ls V-P0 lice (not exposed to H_2_O_2_ for two years). F4 lice were exposed twice to H_2_O_2_, as pre-adult I and pre-adult II (Table 6). When the females became adults, they were either exposed to H_2_O_2_ for a third time immediately before fixation or served as H_2_O_2_-selected control samples without H_2_O_2_ exposure during the adult stage. No differences in the expression levels of *catalase* were apparent between the three groups (statistical analysis could not be performed due to the low sample size in the groups, but the range of values overlapped; see Fig. 6).

The selection of resistant lice (Ls V) with H_2_O_2_ during three generations appears neither to increase significantly the resistance level of the population (EC_50_ values), nor to change *catalase* expression. Even though the EC_50_ values did not increase significantly, if the *catalase* induction hypothesis is correct, one would expect that the H_2_O_2_ exposure would change the expression of that gene in resistant parasites. However, a plausible explanation is that the selected lice were protected from the H_2_O_2_ exposure at the protein level, not needing to regulate the gene expression. It has been shown that the exposure of a shrimp species to a high concentration of H_2_O_2_ can induce *catalase* expression, whereas lower concentrations only alters catalase activity at the protein level [27]. Dawson & Storey [42] showed that post-translational modifications of catalase could regulate the enzyme activity. Exposing resistant lice to 1000 ppm may be considered a “low” concentration for parasites that are able to survive 2500 ppm (Table 6).

## Conclusions

*Catalase* gene expression seems to be induced by H_2_O_2_ exposure. This may pose a challenge for its use as a sole biomarker for resistance, as a test should detect resistant parasites regardless of previous exposure history. Moreover, the amount and activation state of the catalase protein cannot be discarded as part of the resistance mechanism, and lice may not need to modify the gene expression if they are protected at the protein level. The RNAseq study identified several genes differentially expressed when comparing resistant to sensitive lice, but most of them seemed related to a previous H_2_O_2_ exposure. However, five genes were consistently up- or downregulated in resistant lice independently of the H_2_O_2_ exposure, which make them potential good, complementary candidate genes for developing molecular markers for monitoring H_2_O_2_ resistance. The more promising one was *Glp1_v2*, an aquaglyceroporin, that was downregulated in all three groups of resistant lice. Since some aquaporins may serve as a passing channel for H_2_O_2_, lower protein number could reduce the influx or distribution of H_2_O_2_ in the salmon louse, being thus directly involved in the resistance mechanism.

## Supplementary information

**Additional file 1: Script S1.** R-script to identify shared genes differentially expressed in the H_2_O_2_-resistant lice. Shared genes across all the groups (2013, P0 and F2) or between two of the groups (2013 *vs* P0; 2013 *vs* F2; and P0 *vs* F2), separately for up- and downregulated genes.

**Additional file 2: Script S2.** R-script to identify shared genes differentially expressed in the H_2_O_2_-resistant lice. Shared genes across all the groups (2013, P0 and F2) or between two of the groups (2013 *vs* P0; 2013 *vs* F2; and P0 *vs* F2), separately for up- and downregulated genes.

**Additional file 3: Dataset S1.** Complete list of genes differentially expressed in the H_2_O_2_-resistant lice groups (Ls V-2013, Ls V-P0 and Ls F2-R) *versus* the corresponding sensitive groups (Ls A-2013, Ls A-P0 and Ls F2-S), separately for up- and downregulated genes. Genes identified by their ENSEMBL entries. *P*(adj): *P*-value for normalized counts (α = 0.05). **Table S1.** Genes upregulated in Ls V-2013 *vs* Ls A-2013. **Table S2.** Genes downregulated in Ls V-2013 *vs* Ls A-2013.**Table S3.** Genes upregulated in Ls V-P0 *vs* Ls A-P0. **Table S4.** Genes downregulated in Ls V-P0 *vs* Ls A-P0. **Table S5.** Genes upregulated in Ls F2-R *vs* Ls F2-S. **Table S6.** Genes downregulated in Ls F2-R *vs* Ls F2-S.

**Additional file 4: Dataset S2.** Genes differentially expressed shared between H_2_O_2_-resistant lice, separately for up- and downregulated genes. Genes identified by their ENSEMBL entries. **Table S1.** Shared genes downregulated between two resistant groups: 2013 *vs* P0, 2013 *vs* F2 or P0 *vs* F2. **Table S2.** Shared genes downregulated across all the resistant groups: 2013, P0 and F2. **Table S3.** Shared genes upregulated between two resistant groups: 2013 *vs* P0, 2013 *vs* F2 or P0 *vs* F2. **Table S4.** Shared genes upregulated across all the resistant groups: 2013, P0 and F2.

## Data Availability

The datasets generated and analyzed during the current study are available in the Sequence Read Archive (SRA) repository, NCBI, PRJNA636941, http://www.ncbi.nlm.nih.gov/bioproject/636941.

## Abbreviations

cDNA: complementary DNA
CI: confidence interval
EC_50_: compound concentration affecting 50% of the parasites
ERP29: endoplasmic reticulum resident protein 29
Fx: generation of lice
F2: second generation after crossing of Ls A and Ls V lice strains
Glp1_v2, Aqp12L1, Aqp12L2, Glp2, Glp3_v1: several aquaporin types
H_2_O_2_: hydrogen peroxide
Ls A: sensitive *Lepeophtheirus salmonis* strain
Ls V: resistant *Lepeophtheirus salmonis* strain
ppm: parts per million
NA: unannotated protein
qPCR: quantitative polymerase chain reaction
RNA: ribonucleic acid
RNAseq: RNA sequencing
